# RBM8A Promotes Glioblastoma Growth and Invasion Through the Notch/STAT3 Pathway

**DOI:** 10.3389/fonc.2021.736941

**Published:** 2021-11-04

**Authors:** Yan Lin, Lei Wei, Beiquan Hu, Jinyan Zhang, Jiazhang Wei, Zhongrun Qian, Donghua Zou

**Affiliations:** ^1^Department of Medical Oncology, Guangxi Medical University Cancer Hospital, Nanning, China; ^2^Department of Neurology, The Fifth Affiliated Hospital of Guangxi Medical University, Nanning, China; ^3^Department of Neurosurgery, The Fifth Affiliated Hospital of Guangxi Medical University, Nanning, China; ^4^Department of Otolaryngology & Head and Neck, The People’s Hospital of Guangxi Zhuang Autonomous Region, Guangxi Academy of Medical Sciences, Nanning, China; ^5^Department of Neurosurgery, The First Affiliated Hospital of University of Science and Technology of China, Division of Life Sciences and Medicine, Hefei, China

**Keywords:** RBM8A, glioblastoma, prognosis, Notch, stat3

## Abstract

**Background:**

Glioblastoma (GBM) is a prevalent brain malignancy with an extremely poor prognosis, which is attributable to its invasive biological behavior. The RNA-binding motif protein 8A (RBM8A) has different effects on various human cancers. However, the role of RBM8A in GBM progression remains unclear.

**Methods:**

We investigated the expression levels of RBM8A in 94 GBM patients and explored the correlation between RBM8A expression and patient prognosis. Using *in vitro* and *in vivo* assays, combined with GBM sequencing data from the Cancer Genome Atlas (TCGA) and the Chinese Glioma Genome Atlas (CGGA), we examined whether and how RBM8A contributes to GBM progression.

**Results:**

RBM8A was up-regulated in GBM tissues, and its higher expression correlated with worse prognosis. Knockdown of RBM8A inhibited GBM progression and invasion ability both *in vitro* and *in vivo*. On the contrary, overexpression of RBM8A promoted GBM progression and invasion ability. Enrichment analysis of differentially expressed genes in GBM data identified the Notch1/STAT3 network as a potential downstream target of RBM8A, and this was supported by molecular docking studies. Furthermore, we demonstrated that RBM8A regulates the transcriptional activity of CBF1. The γ-secretase inhibitor DAPT significantly reversed RBM8A-enhanced GBM cell proliferation and invasion, and was associated with down-regulation of p-STAT3 and Notch1 protein. Finally, the gene set variance analysis score of genes involved in regulation of the Notch1/STAT3 network by RBM8A showed good diagnostic and prognostic value for GBM.

**Conclusions:**

RBM8A may promote GBM cell proliferation and migration by activating the Notch/STAT3 pathway in GBM cells, suggesting that RBM8A may serve as a potential therapeutic target for the treatment of GBM.

## Background

Glioblastoma multiforme, also known as glioblastoma (GBM), is a highly malignant and prevalent brain tumor associated with extremely poor prognosis (1, 2). GBM tissues have strong invasive potential and do not show an obvious boundary from normal brain tissue. Therefore, it is difficult to remove the tumor completely, and the recurrence rate is quite high. Despite progress in neurosurgery, radiotherapy, and chemotherapy, most GBM patients show poor prognosis, and their five-year survival rate is between 3 and 9% (3). The molecular markers included in diagnostic and classification criteria for central nervous system tumors have recently expanded to include, for example, a mutation in the *IDH* gene, the combined deletion 1p/19q, the histone modification H3K27M, and immunopositivity for the RELA fusion protein (4, 5). Recent studies have found that growth factors and cytokines (GFCKs) play a critical role in tumor invasion (6). Aberrant methylation of gene promoters appears to contribute to a broad variety of cancers, including GBM (7). Recent studies have confirmed the view that GBM proliferation and invasiveness can be increased through a network of gene signaling pathways (8). We know little about the mechanisms and epigenetic variations that promote the development and progression of GBM, and therefore identifying new markers associated with glioblastoma may be useful in diagnosis and treatment.

Abnormal expression of key factors in the nonsense-mediated mRNA decay (NMD) pathway has been associated with various types of cancer, including GBM. The NMD pathway helps maintain homeostasis and regulate cellular functions by eliminating mRNA transcripts that contain premature termination codons, and by degrading normal mRNAs that encode proteins that promote cell growth, migration, and cell survival (9, 10). Loss of NMD function is known to promote tumor growth and invasion (11), suggesting that targeting key factors of the NMD pathway may be effective for cancer treatment (12–14).

One such NMD factor is the RNA-binding motif protein 8A (RBM8A), which serves as a core factor of the exon junction complex (EJC). The EJC acts as a node in post-transcriptional regulatory networks in eukaryotes (15–17). The *RBM8A* gene, located on chromosome 1q21.1, is abundantly expressed in cells, where it shuttles between the cytoplasm and nucleus (17). It helps regulate RNA transcription, translation, cell cycle regulation, and apoptosis (18–21). RBM8A is abnormally expressed in several types of tumors, including cervical cancer, non-small-cell lung carcinoma, myeloma, and hepatocellular carcinoma (22–25). It binds to the transcriptional factor STAT3 to promote its DNA binding and thereby upregulate target genes (26, 27). The function of RBM8A in GBM has not yet been reported.

The Notch signaling pathway is an evolutionarily conserved pathway that plays a key role in cell proliferation, apoptosis, stem cell maintenance, cell fate determination, tissue homeostasis as well as other cell and development processes (28–30). The abnormal regulation of Notch signaling is related to many tumors. Particularly, in the development of brain cancer, Notch1 has been reported to be carcinogenic (31). However, the possible relationship among RBM8A, Notch and STAT3 in the context of brain tumors has not been clearly defined.

In this study, we analyzed the expression of RBM8A in GBM. In addition, the functional impact of RBM8A on GBM progression and its underlying molecular mechanisms were also studied using *in vitro* and *in vivo* assays. A comprehensive regulatory network involving RBM8A and the Notch/STAT3 pathway in GBM was established to explore the potential biological mechanisms of RBM8A. The results of this study may provide novel insights into the role of RBM8A in GBM progression and help identify potential therapeutic targets in the disease.

## Materials and Methods

### Patients

This study was approved by the Ethics Committee of the Fifth Affiliated Hospital of Guangxi Medical University. Written consent was obtained from all patients enrolled in the study, or from their legal guardians. A total of 94 patients (45 males and 49 females) were recruited between January 2005 and December 2013 at the Renji Hospital. All recruited patients had a histologically confirmed diagnosis of GBM, which was independently reviewed by two pathologists and classified based on WHO criteria for tumor grading. Samples of cancerous tissue (n=5) and normal tissues (n=5) were collected from patients.

### Data Processing

GBM data in The Cancer Genome Atlas (TCGA) (https://www.cancer.gov/) were downloaded from the UCSC Xena browser (http://xena.ucsc.edu/public), including gene expression profiles based on Affymetrix Human Genome U133a array platform (Affymetrix; Thermo Fisher Scientific, Waltham, MA, USA) and clinical information. In addition, two glioma data sets (mRNAseq_693 and mRNAseq_325) were obtained from the Chinese Glioma Genome Atlas (http://cgga.org.cn/index.jsp).

### Western Blot Analysis

The expression of RBM8A was analyzed using western blots, which were performed using a rabbit antibody against human RBM8A (14958-1-AP, Proteintech, IL, USA; 1:1000) and mouse antibody against beta-actin (Millipore, MA, USA; 1:10,000) (32). The tissues were lysed with an RIPA buffer [50 mM Tris-HCl (pH 7.5), 150 mM NaCl, 1% Triton X-100, 0.5% Na-deoxycholate] containing protease inhibitors (CompleteMini, Roche, Basel, Switzerland). Samples of the lysates (20-30 μg) were separated on 8-12% SDS-PAGE gels and transferred to polyvinylidene fluoride membranes, which were incubated overnight with primary antibodies at 4°C, then with horseradish peroxidase-conjugated secondary antibody. An ECL kit (catalog no. PI32209, Pierce, IL, USA) was used to detect the bound antibodies.

### Luciferase Reporter Assay to Detect Notch Activation

Notch activation was assessed using the CBF1 luciferase reporter system (Dual-Glo Luciferase Assay System, Promega, USA). The *Renilla* luciferase plasmid pRLTK (Promega), which controls for transfection efficiency, was cotransfected with CBF1-Luciferase reporter plasmid using Lipofectamine 2000 (Thermo Fisher Scientific). Cells were harvested after 24 h in culture, and luciferase activity was determined using the Luciferase Assay System (Promega) and a microplate luminometer (Berthold, Bad Wildbad, Germany).

### Immunohistochemistry

Immunohistochemistry was performed on the paired samples from 94 patients as described (33). Tissue microarray blocks were constructed, and serial sections (4 μm) were obtained from each block. The first slide was stained with hematoxylin and eosin (H&E) to confirm the diagnosis, and subsequent slides were stained appropriately for further analysis.

Tissue microarray slides were deparaffinized, rehydrated, blocked and stained with primary antibody against human RBM8A (14958-1-AP, Proteintech; 1:100). The sections were then stained using a highly sensitive streptavidin-biotin-peroxidase detection system and counterstained with hematoxylin. Negative controls were processed in parallel using pre-immune immunoglobulin instead of primary antibody.

Sections of cancerous tissue from each patient were independently evaluated by two pathologists blinded to clinicopathological information. Immunoreactive staining was quantified in terms of the percentage of positive cells, with a score of 0 meaning that 0% of tumor cells showed positive staining; 1, up to 10% of cells were positive; 2, 11-50%; 3, 51-75%; 4, 75-100%. The intensity of staining was quantified using a four-point scale, where 0 meant negative; 1, weak; 2, moderate; and 3, strong. The two scores for each section were then multiplied together to give a total score ranging from 0 to 12. All patients were classified based on RBM8A expression as negative (total score 0), low (score 1-4), moderate (score 5-8), or high (score 9-12).

### Cell Lines

The human GBM cell lines U87-MG, U251-MG, A172, and T98G were purchased from ATCC and cultured in Dulbecco’s modified Eagle medium (Invitrogen, Carlsbad, CA, USA) supplemented with 10% fetal bovine serum (FBS, Invitrogen), and maintained at 37°C in a humidified atmosphere of 5% carbon dioxide.

### Lentiviral Vector-Mediated RBM8A Knockdown

Lentiviral expression plasmids encoding small hairpin RNA (shRNA) targeting human RBM8A as well as encoding green fluorescent protein (GFP) were constructed by Hanyin (Shanghai, China). Three plasmids were constructed, each with a different RBM8A-shRNA: 5’-AGAGCATTCACAAACTGAA-3’ (RBM8A-KD1), 5’-CATCAGCGTTGACTGGTGT-3’ (RBM8A-KD2), and 5’-GCAACAGGTCTAGGGTTAAGG-3’ (RBM8A-KD3). As a negative control, lentiviral expression plasmid encoding only GFP (GFP-lentivirus) was also prepared. The knockdown efficiency of cells infected with the RBM8A-shRNA-encoding virus was confirmed after 48 h using quantitative real-time polymerase chain reaction (qRT-PCR) and western blots.

Recombinant RBM8A-shRNA lentivirus and negative control (NC) lentivirus were prepared. To obtain stable cell lines showing RBM8A knockdown (RBM8A-KD) and stable NC cell lines, U87-MG and U251-MG cells were seeded in 6-well plates at a density of 2 × 105 cells per well. On the following day, the cells were infected with the virus at a multiplicity of infection of 1 using Polybrene (8 μg/ml; Sigma-Aldrich, IL, USA). GFP expression was confirmed under a fluorescence microscope approximately 72 h after viral infection, and the culture medium was replaced with a selection medium containing puromycin (4 μg/ml). Cells were cultured for a minimum of 14 days. The puromycin-resistant cells were amplified in a medium containing 2 μg/ml puromycin for 7-9 days, then cultured in medium without puromycin.

### Lentivirus-Mediated RBM8A Overexpression

An expression plasmid encoding FLAG-tagged RBM8A was engineering using the pMSCV-IRES-GFP vector (Hanyin), and this plasmid or the corresponding empty vector as NC were transfected into 293T cells. Recombinant retrovirus from these cells was used to infect A172 and T98G cells at a multiplicity of infection of 1, giving RBM8A-overexpressing (RBM8A-OE) cells or NC cells.

### Cell Proliferation Assays

Cell proliferation over a period of five days was examined using the Cell Counting Kit-8 assay (Beyotime, Shanghai, China). Cells (n = 1000) were seeded into 96-well plates. At different time points during 5-day treatment with the γ-secretase inhibitor DAPT, CCK-8 solution (10 μl) was added to each well, and cells were incubated for 2 h at 37°C. Absorbance at 450 nm was measured using a microtiter plate reader (33). This experiment was conducted in triplicate.

A proliferation assay based on 5-ethynyl-2′-deoxyuridine (EdU) was performed according to the manufacturer’s instructions (Invitrogen).

### Transwell Assays

Cells were plated in the upper chamber of Transwell assay inserts (Millipore, Billerica, MA, USA) with or without a Matrigel-coated membrane containing 8-μm pores in serum-free RPMI 1640 medium (200 μl). The inserts were then placed in the bottom chamber of a 24-well plate containing RPMI 1640 with 10% FBS as a chemoattractant. The top layer of the insert was scrubbed after 24 h with a sterile cotton swab to remove any remaining cells. The invading cells on the bottom layer were stained with 0.1% crystal violet, examined, counted, and imaged using digital microscopy. The numbers of cells in five random fields of each chamber were counted and averaged. These assays were conducted in triplicate.

### Xenograft Animal Models

This study followed institutional guidelines as well as the guidelines put forth by the US National Institutes of Health on animal welfare and experimentation. The animal experiments were approved by the Institutional Animal Care and Use Committee of Guangxi Medical University. RBM8A-KD or NC U87-MG cells were intracranially implanted into the corpus striatum of anesthetized 6-week-old athymic nude mice using a stereotactic frame for small animals (David Kopf Instruments, Tujunga, California, USA). Tumor size was monitored by magnetic resonance imaging (MRI).

### Enrichment Analysis

Functional enrichment analysis was performed using the Clusterprofiler package in R (34). In addition, GSEA was carried out using GSEA software, which can be found on the official website (http://software.broadinstitute.org/gsea/index.jsp). Gene sets c5.bp.v7.1.symbols.gmt and c2.cp.kegg.v7.1.symbols.gmt in the MsigDB database were used as the reference gene sets (35). A nominal value of p < 0.05 was considered statistically significant.

### Construction of a Comprehensive Regulatory Network and Molecular Docking

Based on the STRING database (36), a comprehensive regulatory network involving RBM8A in GBM was constructed. We downloaded the PDB files of RBM8A from the Protein Database (https://www.rcsb.org) (37). The three-dimensional structure of CBF1 was predicted using RNA Composer (http://rnacomposer.cs.put.poznan.pl). Molecular docking was performed using Hex8.0.0 software (38), and the results were visualized with Pymol software (39).

### Receiver Operating Characteristic Curve Analysis and Survival Analysis

We extracted the genes involved in the comprehensive network regulated by high RBM8A expression, and calculated the gene set variation analysis (GSVA) scores of these genes using the GSVA package (40). In addition, the receiver operating characteristic (ROC) curve for the GSVA score was analyzed using the pROC package (41), and survival analysis based on the GSVA score was performed using the “survival” package in R (https://CRAN.R-project.org/package=survival).

### Statistical Analyses

Patient survival was calculated from the date of surgery until the date of death or last follow-up. Survival curves for patients whose tumors contained different levels of RBM8A were plotted using the Kaplan-Meier method and compared using the log-rank test. Median survival times and hazard ratios (HRs) were presented with 95% confidence intervals (CIs). All statistical analyses were performed using the R package and SPSS for Windows 17.0 (SPSS, Chicago, IL, USA). Differences were considered significant when the two-tailed P value was less than 0.05.

## Results

### Association of High RBM8A Expression With Poor Prognosis

**Figure 1:**
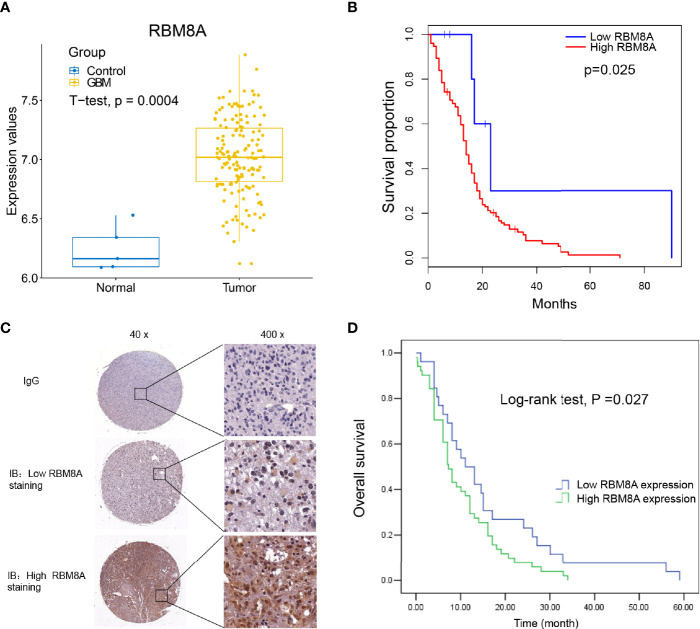
Up-regulated RBM8A expression levels suggest an unfavorable prognosis in GBM. **(A)** RBM8A was up-regulated in GBM based on The Cancer Genome Atlas. **(B)** Kaplan-Meier curves for the GBM samples in TCGA based on optimal gene expression grouping. GBM patients with high expression of RBM8A showed poor prognosis. **(C)** Representative images from immunohistochemical staining of tumor sections from GBM patients. Immunoglobulin (IgG) was used as a negative control. Magnification: 40× or 400×. Representative photographs show low or high RBM8A expression in GBM tissues. **(D)** Kaplan-Meier curves of overall survival for patients whose tumors showed low or high RBM8A expression.

First, the expression of RBM8A in GBM was analyzed using the TCGA database. Compared with control samples, RBM8A was significantly higher in GBM, suggesting that RBM8A may play an important role in GBM (**Figure 1A**). Even more interesting is that GBM patients with high expression of RBM8A in TCGA had poor overall survival, based on the optimal gene expression grouping threshold (**Figure 1B**). RBM8A expression in GBM specimens from the 94 patients was examined using immunostaining (**Figure 1C**). The mean age of the 94 included patients at diagnosis was 49.38 ± 15.87 years (range, 13-85 years). RBM8A localized primarily to the nucleus and we found that a large proportion of patients with low-grade tumors (78.57%) had low RBM8A expression, while those with high-grade tumors (66.25%) had high RBM8A expression (**Table 1**). Follow-up data lasting a mean of 14.74 ± 13.13 months (range, 0.03-59 months) were available for 76 patients, who were therefore included in the survival analyses.

**Table 1:** Clinicopathological characteristics of patients stratified by RBM8A expression level.

Characteristic	All patients	RBM8A expression		P value
	(n = 94)	low (n = 38)	high (n = 56)	
**Age (year)**				
< 55	43 (45.74%)	22 (51.16%)	21 (48.84%)	0.201
≥ 55	51 (54.26%)	16 (31.37%)	35 (68.63%)	
**Gender**				
Male	45 (47.87%)	16 (35.56%)	29 (64.44%)	0.362
Female	49 (52.13%)	22 (44.90%)	27 (55.10%)	
**WHO grade**				
Early (I+II)	14 (14.89%)	11 (78.57%)	3 (21.43%)	**0.001**
Late (III+IV)	80 (85.11%)	27 (33.75%)	53 (66.25%)	
**Tumor location**				
Frontal	37 (39.36%)	14 (37.84%)	23 (62.16%)	0.684
Non-frontal	57 (60.64%)	24 (42.11%)	33 (57.89%)	

Values are n (%) unless otherwise noted.

We compared overall survival between the patients showing relatively low and high expression levels. Among the 76 patients with follow-up data, those with low nuclear levels of RBM8A expression in tumor tissues (n = 26) exhibited significantly longer overall survival than those with high nuclear RBM8A expression (n = 50; **Figure 1D**). The median survival time was 11.0 months (95%CI 6.00-15.99) among patients with low RBM8A expression but 7.0 months (95%CI 5.46-8.54) among those with high RBM8A expression (**Table 2**). The log-rank test revealed that patients with high RBM8A expression had significantly shorter overall survival time (χ2 = 4.884, P = 0.27; **Table 2**).

**Table 2:** Overall survival time of patients stratified by RBM8A expression level.

Group	n	Median time, mos.	95% CI	Chi-squared (χ^2^)	P value
Low RBM8A expression	26	11.0	6.00-15.99	4.884	0.027
High RBM8A expression	50	7.0	5.46-8.54		
All patients	76	8.0	5.34-10.66		

### RBM8A Knockdown Reduces GBM Cell Proliferation and Invasion *In Vitro*

**Figure 2:**
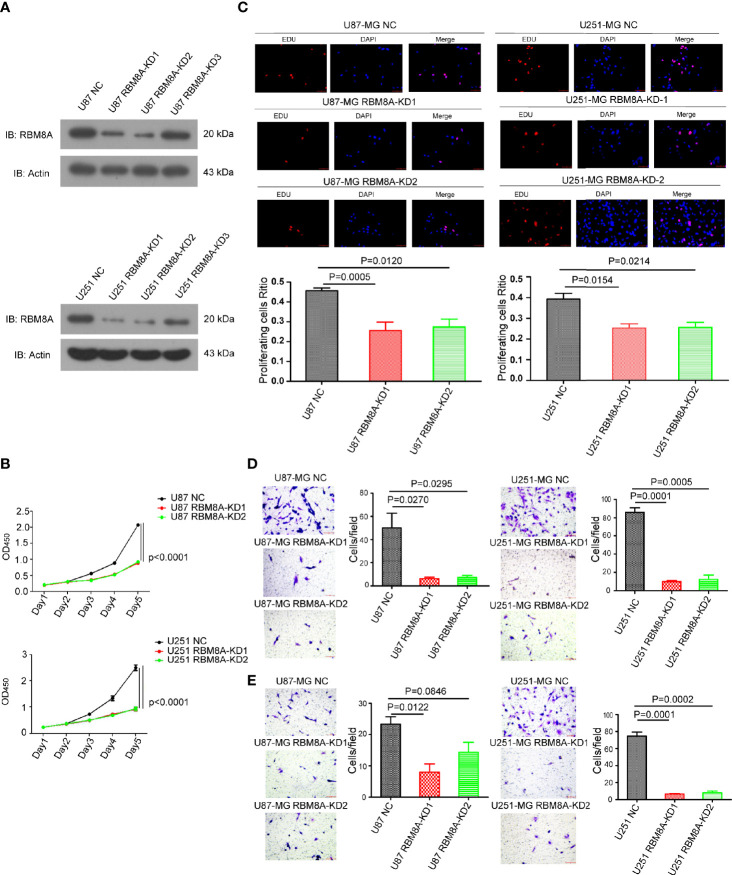
Effects of RBM8A knockdown on proliferation of U87-MG and U251-MG cells and their invasive activity. **(A)** Expression of RBM8A in U87 or U251-MG cells transformed with control lentivirus (NC) or lentivirus expressing the knockdown-1 (KD1) or knockdown-2 (KD2) small hairpin RNA against the RBM8A gene were determined by western-blotting. **(B)** Cell proliferation was analyzed using the CCK8 assay as indicated. **(C)** Cell proliferation was also measured using the EdU assay. Representative fluorescence micrographs of the different cell cultures are shown. Immunostaining levels are quantified in the plots below. **(D)** Transwell analysis of migration, using two-chamber wells without a Matrigel-coated insert. Magnification, 200×. **(E)** Transwell analysis of invasion, using two-chamber wells with a Matrigel-coated insert. All micrographs are shown at 200× magnification.

To understand the function of RBM8A in GBM progression, we examined the expression of RBM8A in GBM cell lines using western blotting. RBM8A expression was higher in U87-MG and U251-MG cells (**Supplementary Figure S1**). After effectively knocking down RBM8A in the U87-MG and U251-MG cells (**Figure 2A**), we assessed the function of RBM8A in GBM cell proliferation and invasion. CCK8 and EdU assays indicated that the suppression of RBM8A reduced the ability of GBM cells to proliferate (**Figures 2B, C**). Transwell assays demonstrated that RBM8A knockdown significantly suppressed migration and invasion by GBM cells (**Figures 2D, E**). These findings suggest that RBM8A expression plays a crucial role in promoting the proliferation and invasive potential of GBM cells.

### RBM8A Overexpression Enhances GBM Cell Proliferation and Invasion *In Vitro*

**Figure 3:**
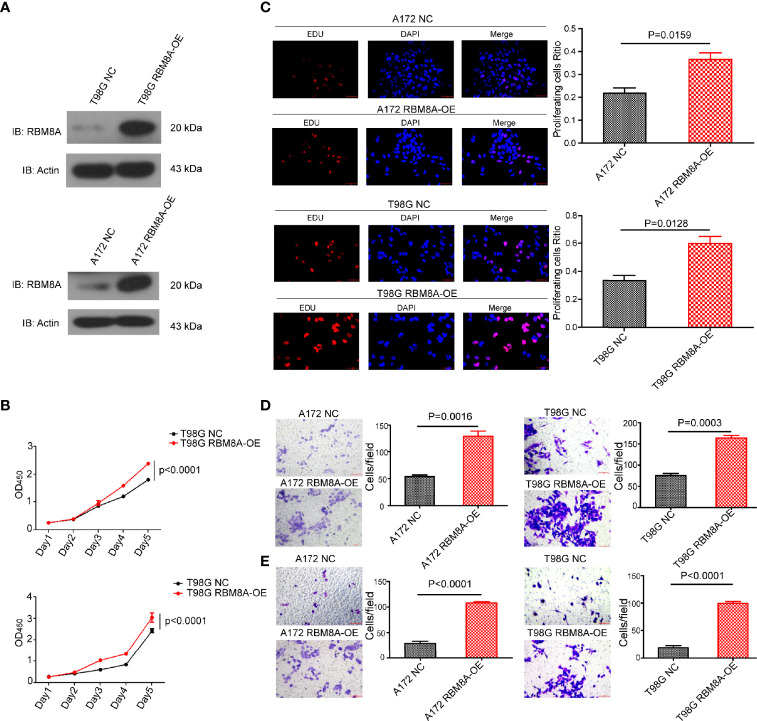
Effects of RBM8A overexpression on proliferation of A172 and T98G GBM cells and their invasive activity. **(A)** Expression of RBM8A in T98G cells transformed with control lentivirus (NC) or lentivirus expressing RBM8A (RBM8A-OE) was assessed by western blotting. **(B)** Cell proliferation was analyzed using the CCK8 assay as indicated. **(C)** Cell proliferation was also measured using the EdU assay. Representative fluorescence micrographs of the different cell cultures are shown. **(D)** Transwell analysis with or without RBM8A overexpression. **(E)** Matrigel-Transwell analysis with or without RBM8A overexpression. All micrographs are shown at 200× magnification.

To verify and extend our results obtained with RBM8A knockdown, we overexpressed RBM8A in A172 and T98G cells (**Figure 3A**). Overexpression enhanced GBM cell proliferation (**Figures 3B, C**), as well as migration and invasion (**Figures 3D, E**). These findings provide evidence that elevated RBM8A expression promotes GBM cell tumorigenesis and invasion.

### The Notch/STAT3 Pathway Mediates the Pro-Oncogenic Function of RBM8A in GBM Cells

**Figure 4:**
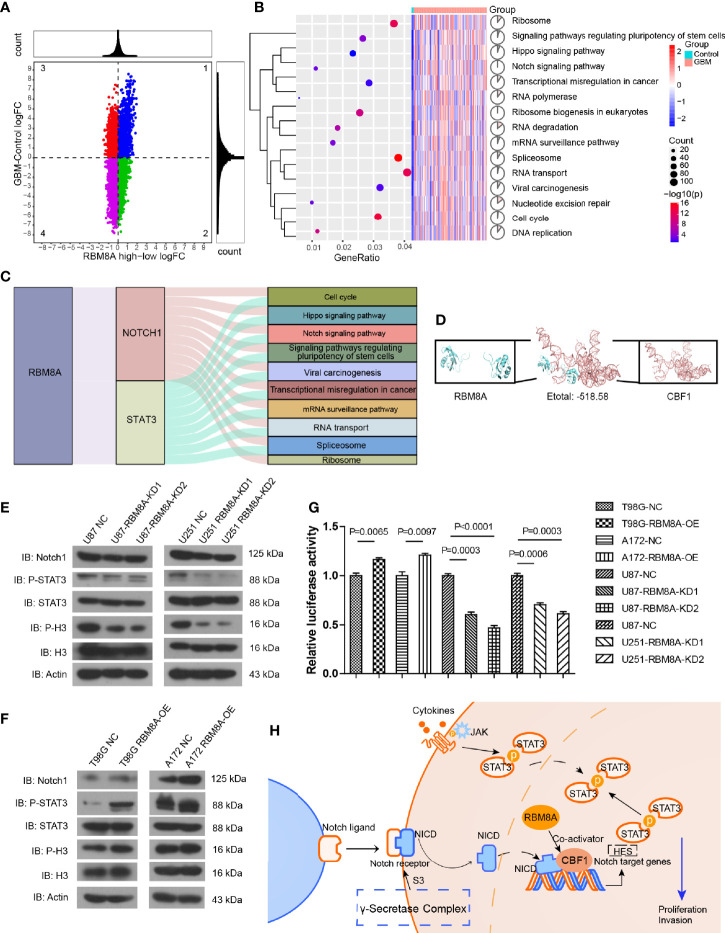
Effects of RBM8A expression on the Notch/STAT3 pathway in GBM cells. **(A)** Quadrant plot. The genes in quadrant 2 were upregulated genes consistent with RBM8A expression. **(B)** Analysis of Kyoto Encyclopedia of Genes and Genomes (KEGG) pathway enrichment was performed on the consistently up-regulated genes. **(C)** RBM8A affects biological pathways by regulating Notch1 and STAT3. **(D)** Molecular docking of RBM8A and CBF1. **(E)** Western blot analysis of Notch1, phosphorylated STAT3 (p-STAT3), total STAT3 (STAT3), phosphorylated H3 (p-H3), total H3 (H3), and actin in U87-MG and U251-MG cells transformed with negative control (NC) lentivirus or with lentivirus encoding RBM8A-KD1 or -KD2. **(F)** Western blot analysis of the same proteins in A172 and T98G cells transformed with NC lentivirus or lentivirus encoding RBM8A (RBM8A-OE). **(G)** Luciferase reporter assay of RBM8A-induced activation of CBF1 in GBM cells. **(H)** RBM8A regulates the Notch/STAT3 signaling pathway by targeting CBF1, which may be a mechanism by which RBM8A contributes to GBM development.

To further explore the effect of high expression of RBM8A on GBM, we performed differential expression analysis between the GBM and control samples, as well as between groups expressing high or low RBM8A levels. Genes consistent with high RBM8A expression were identified as differentially expressed genes (DEGs) (**Figure 4A**). DEGs may be affected by the high expression of RBM8A and may be involved in the ability of RBM8A to promote tumor growth and invasion. These DEGs were involved mainly in the cell cycle, Notch signaling pathway and Hippo signaling pathway (**Figure 4B**).

Based on the STRING database, we identified the pathway genes interacting with Notch1 and STAT3. RBM8A may regulate Notch1 and STAT3, and then regulate pathway genes to affect the occurrence and development of GBM (**Figure 4C**). Molecular docking led to negative docking energies (**Figure 4D**), suggesting that RBM8A can bind the genes whose transcription is regulated by C promoter-binding factor 1 (CBF1).

We verified these bioinformatics analyses by Western blotting for levels of Notch, phospho-STAT3, and phospho-H3 in cells in which RBM8A was knocked down. We observed decreased levels of all three proteins (**Figure 4E**), while RBM8A overexpression had the opposite effects (**Figure 4F**). CBF1 interacts with the Notch1 receptor to activate the Notch signaling pathway (42). Consistent with this, we found that overexpression of RBM8A showed significantly stronger activation of the CBF1 luciferase reporter in T98G and A172 cells than in the controls. In contrast, knocking down RBM8A in U87 and U251 cells weakened activation of the CBF1 luciferase reporter (**Figure 4G**). These results suggest that RBM8A is sufficient to activate the Notch signaling pathway. Therefore, we propose that RBM8A promotes the proliferation and migration of GBM cells by activating the Notch/STAT3 pathway (**Figure 4H**).

We integrated GBM data from TCGA and CGGA. Then, we extracted genes involved in a mechanism through which RBM8A activated the Notch/STAT3 pathway to promote proliferation and migration of GBM cells. We calculated a GSVA score to explore whether these genes might have diagnostic or prognostic potential in GBM. Survival analysis showed that patients with high GSVA scores had poor overall survival (**Supplementary Figure S2A**). In addition, the GSVA score showed good diagnostic accuracy (**Supplementary Figure S2B**).

### Involvement of the Notch/STAT3 Pathway in RBM8A-Mediated GBM Cell Proliferation and Invasion

**Figure 5:**
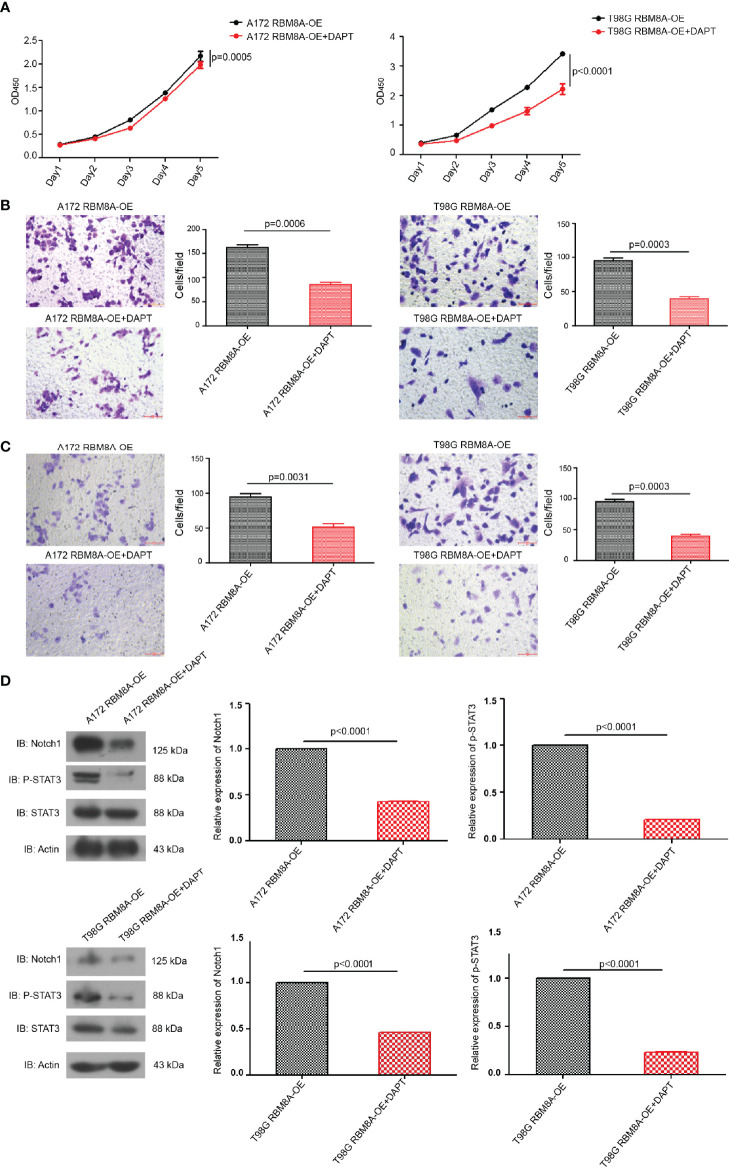
Involvement of the Notch/STAT3 pathway in RBM8A-mediated GBM cell proliferation and invasion. **(A)** Cell proliferation were analyzed in A172 or T98G cells overexpressing RBM8A (OE) in the presence or absence of the γ-secretase inhibitor DAPT using the CCK8 assay. **(B)** Transwell analysis with or without DAPT. **(C)** Matrigel-Transwell analysis with or without DAPT. **(D)** Western blot analysis of Notch1, phospho-STAT3, total STAT3, and actin after incubation with or without DAPT. All micrographs are shown at a magnification of 200×.

Suppression of the Notch pathway in A172 or T98G cells using the γ-secretase inhibitor DAPT significantly reversed RBM8A-enhanced proliferation and invasion (**Figures 5A**–**C**), and this reversal was associated with down-regulation of p-STAT3 and Notch1 protein (**Figure 5D**). These results provide support for the idea that the Notch/STAT3 pathway mediates the pro-oncogenic function of RBM8A in glioblastoma cells.

### RBM8A Knockdown Slows GBM Progression *In Vivo*

**Figure 6:**
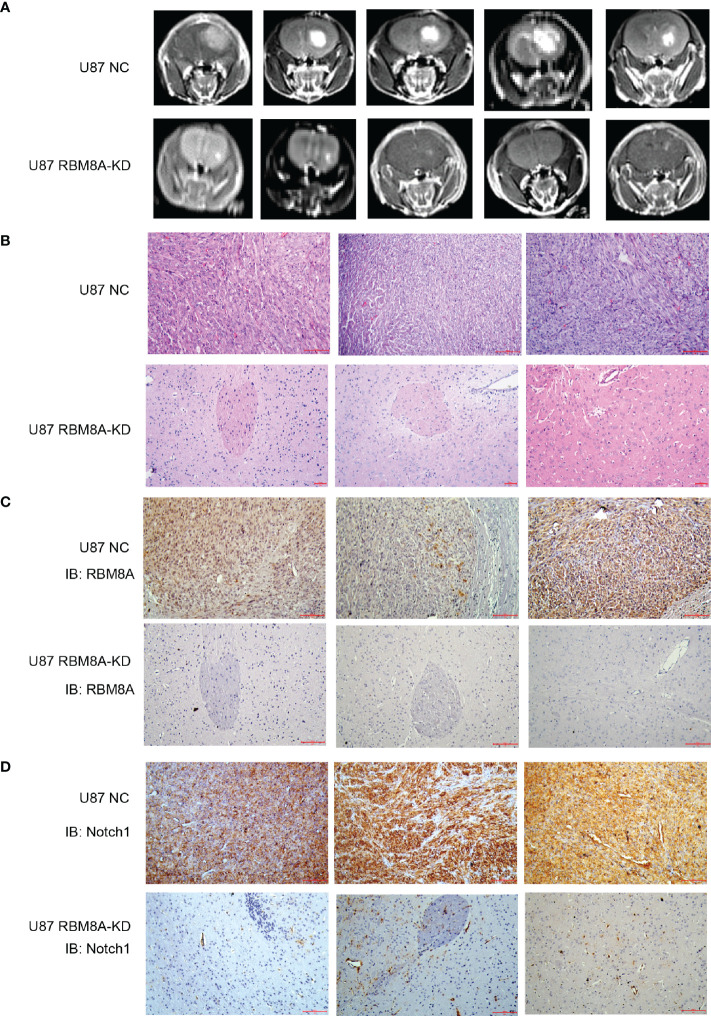
Effects of RBM8A reduction on GBM progression *in vivo*. **(A)** Nude mice were intracranially injected with U87-MG cells transformed with empty lentivirus (NC) or lentivirus encoding short hairpin RBM8A-KD1. At 6 weeks after injection, animals were examined by MRI. **(B)** Representative photomicrographs of tumor sections stained with hematoxylin-eosin (H&E). **(C)** Immunohistochemistry of tumor tissues stained with antibody against RBM8A. **(D)** Immunohistochemistry of tumor tissues stained with antibody against Notch1.

To examine how these effects of RBM8A on GBM cells *in vitro* may translate to clinical phenotypes, we injected U87-MG cells stably expressing RBM8A-KD1 shRNA or no shRNA intracranially into female nude mice and monitored GBM progression. By six weeks, mice inoculated with NC cells developed larger tumors than those inoculated with RBM8A-KD1 cells (**Figures 6A**–**C**). Interestingly, RBM8A knockdown also dramatically reduced the levels of Notch1 protein in tissues (**Figure 6D**). Tumor growth was much slower in the RBM8A-KD group than in the control group, with average tumor diameter 0.25 ± 0.15 mm^3^ in RBM8A-KD animals and 11.92 ± 4.98 mm^3^ in NC animals (p=001; n=5 per group; **Supplementary Figure S3**). These results are consistent with the *in vitro* evidence that RBM8A contributes to GBM progression.

## Discussion

In this study, we found that RBM8A was highly expressed in GBM, and then we explored the effect of RBM8A expression level on the development of GBM. We found that high expression of RBM8A is associated with increased GBM cell growth and invasion, which in turn is associated with shorter overall survival. We further explored the effect of genes consistent with high RBM8A on GBM. We were surprised to find that most of these genes were related to tumorigenesis and tumor development through the cell cycle, Notch signaling pathway, and Hippo signaling pathway, which regulate the pluripotency of stem cells and viral carcinogenesis. We propose that RBM8A may promote GBM cell growth and invasion by regulating Notch1 and STAT3, and here we present *in vitro* and *in vivo* evidence for this proposal. Moreover, we demonstrated that the γ-secretase inhibitor DAPT significantly reverses RBM8A-enhanced GBM cell proliferation and invasion, and this reversal is associated with down-regulation of p-STAT3 and Notch1 protein. Therefore, we hypothesize that RBM8A promotes GBM cell proliferation and migration by activating the Notch/STAT3 pathway, which therefore may be a therapeutic target.

Tumor cells show alterations in normal post-transcriptional regulatory pathways, allowing them to better adapt to the microenvironment (43). The NMD pathway is one such example, so investigating the abnormal function of NMD genes in tumors such as GBM may identify effective treatment strategies (44). In this study, we found GBM to be associated with upregulation of RBM8A, one of the key factors of the NMD pathway. Higher RBM8A expression was also associated with worse prognosis of GBM patients.

Abnormal expression of RBM8A has been observed in many types of cancer, including cervical cancer, non-small-cell lung carcinoma, myeloma, gastric cancer, and hepatocellular carcinomas (23–26). Knocking down RBM8A in lung adenocarcinoma cells arrested the cell cycle, inhibited cell proliferation, activated caspases 3 and 7, and increased the proportion of apoptotic cells containing abnormal centrosomes (19). High expression of RBM8A can also down-regulate splicing variants of multiple pro-apoptotic genes, such as Bcl-X, which inhibits apoptosis of prostate and cervical cancer cells (45). In liver cancer tissues, elevated expression of RBM8A promotes cell proliferation, inhibits apoptosis, and induces the epithelial-mesenchymal transition of tumor cells; higher expression also correlates with worse prognosis (23). Consistent with these studies in other cancers, our study on GBM showed RBM8A overexpression to enhance proliferation and invasion *in vivo* and *in vitro*.

Furthermore, our results suggest that the oncogenic effects of RBM8A in GBM involve activation of the STAT3/Notch pathway. The evolutionarily conserved Notch signaling pathway influences cell growth, survival, apoptosis, migration, and invasion, and it appears to be involved in various human malignancies, including GBM. Notch1 expression positively correlates with glioma progression, and high expression of Notch1 is an independent predictor of low survival rates in patients with gliomas (46). Notch1 knockdown in glioma cells increases cell death, reduces proliferation and arrests the cell cycle (47); it also inhibits growth and invasion of GBM cells (48). Mice with intracranial U251-MG xenografts die earlier when the tumor expresses normal levels of Notch1 than when Notch1 is knocked down (47). Future studies should examine upstream regulators and downstream targets of the Notch pathway in GBM in order to clarify its pathogenesis and identify additional therapeutic targets.

We further explored the mechanistic genes linking RBM8A and the Notch/STAT3 pathway. We found that the GSVA score for these genes has good diagnostic efficacy for GBM, and that higher score is associated with worse prognosis.

Future work should also examine how the ability of RBM8A to activate STAT3/Notch relates to the other functions of RBM8A within the EJC (15, 24), which include downregulating the pro-apoptotic factor Bcl-x in colon and prostate cancer cells and downregulating p53 in a range of tumor cells (45, 49). RBM8A also functions independently of the EJC, such as when it regulates nucleoproteins and is itself phosphorylated (50, 51).

## Conclusion

Based on the association observed in our study between RBM8A expression levels and tumor progression, we conclude that RBM8A may function as an independent prognostic factor and therapeutic target in the management of GBM. Our findings highlight the complexity of roles of RBM8A in tumor proliferation, which requires further investigation, which in turn may uncover additional prognostic biomarkers or therapeutic targets in the management of GBM. However, our study was limited by the experimental models used, and it was only a preliminary exploration of potential molecular interactions. Future research should include the establishment of knockout mouse models to verify whether RBM8A activates the Notch/STAT3 pathway in the development of GBM.

## Data Availability Statement

The datasets presented in this study can be found in online repositories. The names of the repository/repositories and accession number(s) can be found in the article/**Supplementary Material**.

## Ethics Statement

The studies involving human participants were reviewed and approved by The Fifth Affiliated Hospital of Guangxi Medical University. The patients/participants provided their written informed consent to participate in this study. Written informed consent was obtained from the individual(s) for the publication of any potentially identifiable images or data included in this article.

## Author Contributions

YL, LW, ZQ, JW, and DZ conceived and designed the study. DZ, YL, ZQ, JZ, and JW performed the experiments. YL, ZQ, JZ, BH, LW, JW, and DZ analyzed the data, prepared figures and tables, and wrote the manuscript. All authors contributed to the article and approved the submitted version.

## Funding

This study was supported by the National Natural Science Foundation of China (81803041), the High-Level Medical Expert Training Program of Guangxi “139” Plan Funding (G201903049), and the Nanning Excellent Young Scientist Program and the Guangxi Beibu Gulf Economic Zone Major Talent Program (RC20190103).

## Conflict of Interest

The authors declare that the research was conducted in the absence of any commercial or financial relationships that could be construed as a potential conflict of interest.

## Publisher’s Note

All claims expressed in this article are solely those of the authors and do not necessarily represent those of their affiliated organizations, or those of the publisher, the editors and the reviewers. Any product that may be evaluated in this article, or claim that may be made by its manufacturer, is not guaranteed or endorsed by the publisher.

## Supplementary Material

Supplementary Figure S1 RBM8A expression levels in GBM cell lines. Levels of RBM8A mRNA in wild-type U87-MG, U251-MG, T98G and A172 cells.

Supplementary Figure S2 Diagnostic and prognostic value of the GSVA score based on GBM data from TCGA and CGGA. **(A)** Receiver operating characteristic curve of the GSVA score. **(B)** Overall survival curves based on GSVA score.

Supplementary Figure S3 Tumor volume in each group. Data are presented as mean ± SD, and differences were assessed for significance using the two-tailed Student’s *t* test.

## Abbreviations

CBF1, C promoter-binding factor 1; CGGA, Chinese glioma genome map; CI, confidence interval; DEGs, differentially expressed genes; EdU, 5-ethynyl-2′-deoxyuridine; GBM, glioblastoma; GFCKs, growth factors and cytokines; GFP, green fluorescent protein; GSVA, gene set variance analysis; HR, hazard ratio; IgG, immunoglobulin; KD, knockdown; KEGG, Kyoto Encyclopedia of Genes and Genomes; NC, negative control; NMD, nonsense-mediated mRNA decay; MRI, magnetic resonance imaging; OE, overexpressing; PDB, Protein Database; qRT-PCR, quantitative real-time polymerase chain reaction; RBM8A, RNA-binding motif protein 8A; ROC, operator characteristic curve; shRNA, short hairpin RNA; SD, standard deviation; TCGA, The Cancer Genome Atlas.
