# Supplementary figures and images for: RBM8A Promotes Glioblastoma Growth and Invasion Through the Notch/STAT3 Pathway
Source: Front Oncol. 2021 Nov 4;11:736941. doi: 10.3389/fonc.2021.736941 (PMC8600138; doi:10.3389/fonc.2021.736941)

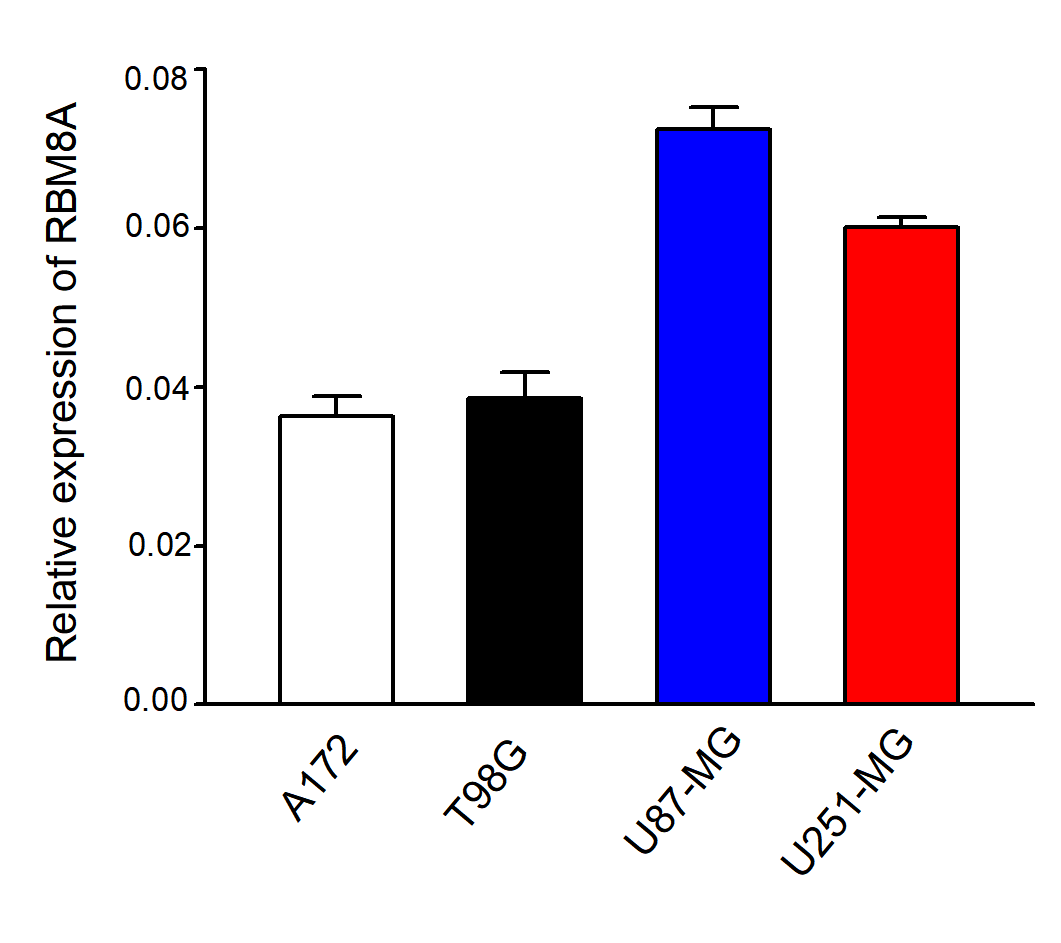

Supplement: Supplementary Figure S1 — RBM8A expression levels in GBM cell lines. Levels of RBM8A mRNA in wild-type U87-MG, U251-MG, T98G and A172 cells. [file Image_1.tif]

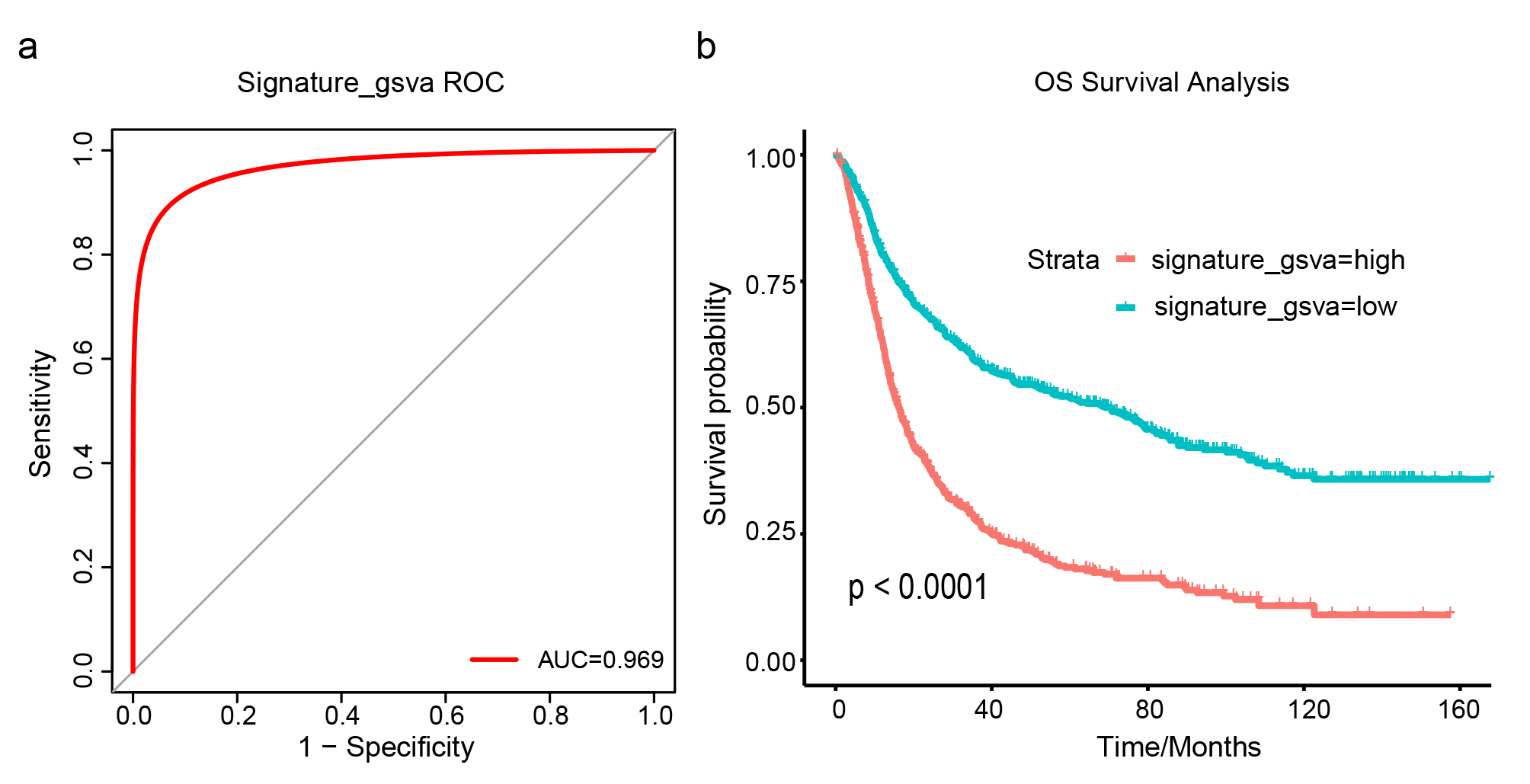

Supplement: Supplementary Figure S2 — Diagnostic and prognostic value of the GSVA score based on GBM data from TCGA and CGGA. (A) Receiver operating characteristic curve of the GSVA score. (B) Overall survival curves based on GSVA score. [file Image_2.tif]

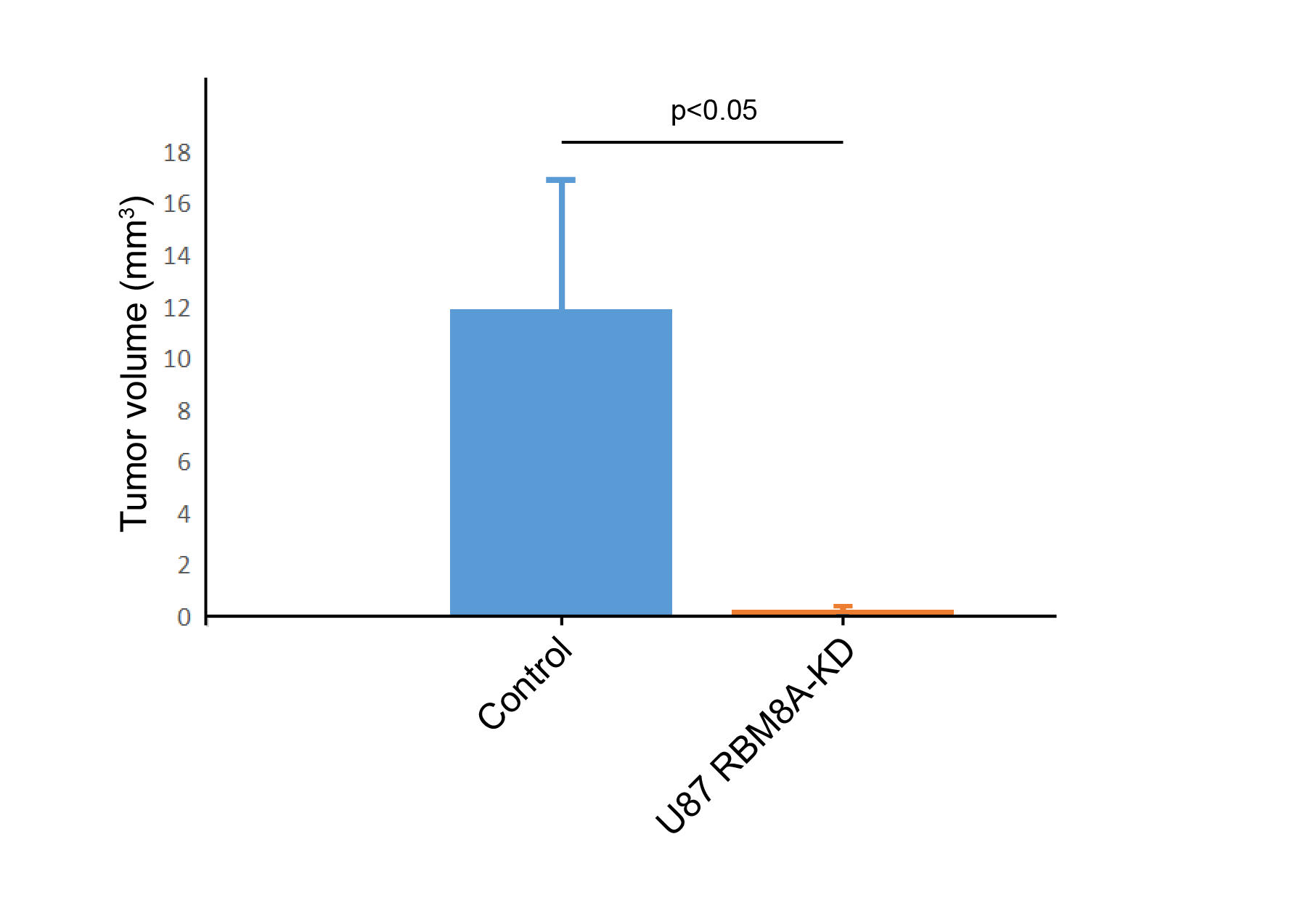

Supplement: Supplementary Figure S3 — Tumor volume in each group. Data are presented as mean ± SD, and differences were assessed for significance using the two-tailed Student’s t test. [file Image_3.png]
